# A modified UTAUT model for acceptance to use telemedicine services and its predictors among healthcare professionals at public hospitals in North Shewa Zone of Oromia Regional State, Ethiopia

**DOI:** 10.3389/fdgth.2025.1469365

**Published:** 2025-06-03

**Authors:** Debela Tsegaye Hailu, Mequannint Sharew Melaku, Solomon Abuhay Abebe, Agmasie Damtew Walle, Kefyalew Naniye Tilahun, Kassahun Dessie Gashu

**Affiliations:** ^1^ Department of Health Informatics, School of Public Health, Institute of Health, Bule Hora University, Bule Hora, Ethiopia; ^2^ Department of Health Informatics, College of Medicine and Health Sciences, Institute of Public Health, University of Gondar, Gondar, Ethiopia; ^3^ Department of Health Informatics, College of Medicine and Health Science, Hawassa University, Hawassa, Ethiopia; ^4^ Department of Health Informatics, College of Health Science, Mettu University, Mettu, Ethiopia; ^5^ Department of Public Health, College of Medicine and Health Science, Ambo University, Ambo, Ethiopia

**Keywords:** acceptance, telemedicine, predictors, healthcare professionals, UTAUT

## Abstract

**Introduction:**

The shortage of healthcare professionals, long waiting time for treatment, inadequate transportation, and hard-to-reach geographical locations remained challenging in the healthcare service delivery in resource-limited settings. To overcome these challenges, healthcare providers are looking to use telemedicine technologies as an alternative solution. However, user resistance has consistently been identified as a major obstacle to the successful implementation of telemedicine. Thus, this study aimed to assess acceptance to use telemedicine services and its predictors among healthcare professionals at public hospitals in the North Shewa Zone of Oromia Regional State, Ethiopia.

**Method:**

A cross-sectional study design was employed among a total of 627 healthcare professionals working at public hospitals in the North Shewa Zone from 3 April to 1 May 2023. The study participants were selected using simple random sampling techniques. A questionnaire, which is adapted from the original instrument developed by Venkatesh et al.'s study and several studies regarding the UTAUT model was used. Data were collected using a self-administered structured questionnaire in English version. The descriptive statistics were estimated using the SPSS version 25, and structural equation modeling analysis was employed using AMOS V.21 software.

**Results:**

In this study, 601 (95.85% response rate) study subjects participated. The study has shown that 315 (52.4%) (95% CI: 48.3–56.5) of the participants accepted to use telemedicine in their routine healthcare services. Performance expectancy (*β* = 0.184, *p* = 0.001), effort expectancy (*β* = 0.183, *p* < 0.001), facilitating conditions (*β* = 0.249, *p* < 0.001), and digital literacy (*β* = 0.403, *p* < 0.001) had a significant positive effect on the acceptance to use telemedicine services. Age was used to moderate facilitating conditions (*β* = 0.400, *p* < 0.001) and digital literacy (*β* = 0.598, *p* < 0.001) in relation to acceptance to use telemedicine services.

**Conclusion:**

The healthcare professionals' acceptance to use the offered telemedicine services was promising for the future. Additionally, our research found significant effects between healthcare professionals' acceptance to use telemedicine services with the predictors except social influence. Facilitating conditions and digital literacy with acceptance to use were moderated by age. Thus, the health facility should strengthen its telemedicine technology by raising awareness of its usefulness and ease of use.

## Introduction

Over the past few decades, digitization in the healthcare industry has evolved drastically with online resources and mobile applications now playing a significant role in healthcare (1). Healthcare organizations are under pressure to incorporate new scientific evidence into practice without compromising quality, access, or equity (2, 3). Information communication and technology (ICT) in healthcare service delivery is becoming more recognized for its potential benefits, with numerous small- and large-scale information system projects that have been initiated throughout African nations (4). One such ICT is telemedicine, which has been considered a necessary measure to alleviate the shortfall of skilled medical specialists in developing countries and discovered to be a quick solution to the shortage of health workers (5).

According to the definition given by the World Health Organization, telemedicine is defined as “the provision of health services, where distance is a critical factor, by all health professionals who use information and communication technologies (ICT) for valid exchange of information for the diagnosis, treatment, and prevention of diseases and injuries, research and evaluation, and for continuing education of healthcare providers, all in the interest of advancing the health of individuals and their communities” (6). Therefore, as it has been described in this definition, the promising potential of telemedicine is an ideal solution that attracts the attention of low-income countries struggling with health facilities and care provider shortages (7).

The World Health Organization (WHO) estimates that 57 countries worldwide have a critical shortage of health workers, with 36 of these countries in sub-Saharan Africa (8), indicating that consumers cannot get adequate and timely healthcare services due to the limited number of healthcare professionals (9). Health workforce shortages continue to be most severe in sub-Saharan African countries, which together bear 24% of the world's disease burden today (8). Ethiopia is one of the countries with a very low health workforce density, which is 0.96 per 1,000 population (9). This is much below five times the minimum level of 4.45 per 1,000 population specified by the WHO to reach the SDG health targets (10), and such shortages have resulted in fragmentation of care delivery (11).

Access to healthcare services is still an issue in Ethiopia, especially in remote or rural areas, due to the high population growth rate, increased demand for healthcare, slow economic growth, rising health costs, inadequate transportation, hard-to-reach geographical locations, and the scarcity of medical specialists, which forced patients to travel long distances to find specialists (12). To overcome this challenge, healthcare providers are looking to use telemedicine as an alternative solution (13). An option to allow access to healthcare services in a developing country such as Ethiopia is implementing telemedicine services and ensuring sustainability (12). Telemedicine is a growing field that can offer professional help over the internet via an app, email, video conferencing, online chat, or phone call (14).

In line with this, Salale University Comprehensive Specialized Hospital considers the potential of using telemedicine services currently in the North Shewa Zone to provide mentorship for local healthcare providers and also bridge the gap between communities and clinicians. The societies in Salale and its surroundings can call the Salale Digital Telemedicine Center (9824) and access the telemedicine services, most especially in general health education, non-communicable disease education, and treatment services; prescriptions; dermatology services; radiology services; ophthalmology services; dietary counseling; mental health counseling; maternal and child health counseling; and treatment services. However, many of such technology systems were not being used to their full potential due to factors such as project management failure, lack of post-implementation sustainability, user resistance, organizational culture, and national culture (15). The slow adoption of telemedicine in many low- and middle-income countries (LMICs) is related to factors such as insufficient infrastructure, slow acceptance, inadequate technological equipment, scarce financial resources, and inadequate skilled human resources (16, 17). Significant obstacles to the adoption of telemedicine may arise from patients' and healthcare providers' poor levels of digital literacy (DL), particularly in remote or low-resource areas (18). A number of issues, including ICT policies, intersectoral collaboration, e-health laws and regulations, telemedicine service-facilitating strategies, integrated supportive supervision on telehealth adoption, and ICT equipment, are associated with the diffusion of telemedicine in most sub-Saharan African nations, particularly Ethiopia (12).

However, user resistance has consistently been identified as a major obstacle to the successful implementation of telemedicine and system implementations, particularly serious in healthcare settings (19, 20). To successfully implement any telehealth services into current health systems, assessing their acceptance is a necessary and preliminary step (14).

Based on the lack of evidence of telemedicine acceptance by healthcare professionals, especially concerning the Salale Digital Telemedicine Center scenario, this study aimed to assess the acceptance to use (ATU) telemedicine services among healthcare professionals and to identify key predictors for facilitating and inhibiting telemedicine service acceptance through the Unified Theory of Acceptance and Use of Technology (UTAUT) model. This finding would be particularly important for the Ministry of Health (MOH), the regional health bureau, Salale University's specialized comprehensive hospital, policymakers, and future research, especially in Ethiopia.

## The theoretical background of the model

The Unified Theory of Acceptance and Use of Technology (UTAUT) is still valid and is used to predict the acceptance behavior of remote care technologies by health professionals (21). The UTAUT model has been developed from a review, synthesis, and validation of eight theories/models of technology use including the theory of reasoned action (TRA), social cognitive theory (SCT), technology acceptance model (TAM), theory of planned behavior (TPB), motivational model, model of PC utilization (MPCU), combined TAM and TPB (C-TAM-TPB) and innovation diffusion theory (IDT), to propose a unified view of technology acceptance (22, 23). For our study, we chose the Unified Theory of Acceptance and Use of Technology (UTAUT) because it is the most comprehensive framework for understanding technology acceptance (23, 24). Several key reasons guided this decision. Firstly, the model has been proven to effectively predict the probability of customers intending to use telemedicine system services (25–28), which is directly relevant to our research focus. Secondly, the UTAUT model has been widely applied in the e-health domain to explore factors influencing the acceptance of telemedicine services (25, 29).

In our study, we assessed healthcare professionals' acceptance to use telemedicine services using the constructs of the modified UTAUT model, as it offers superior predictive power from an individual or consumer perspective (23). This approach ensures a comprehensive evaluation of healthcare information system (HIS) intentions and actual usage. Notably, UTAUT studies are not limited to consumer users alone. Researchers have applied UTAUT to examine various user types, including citizens adopting telemedicine services (30), and nurses’ willingness to embrace home telehealth technologies (31).

Additionally, emphasizing telemedicine as an integral part of health service systems in resource-limited settings such as Ethiopia has several benefits. The implication of using the UTAUT model to implement telemedicine systems in Ethiopia has allowed healthcare providers to convert patient health records into digital formats, enhance the management and quality of data, improve the workflow of supply chain management, make better clinical decisions by exchanging real-time patient data, improve interoperability, efficiently store and share electronic health information, boost customer satisfaction, and enhance the quality of care (32–34). The UTAUT model proposes four predictors, performance expectancy (PE), effort expectancy (EE), social influence (SI), and facilitating conditions (FC), that explain behavioral intention (24). UTAUT identifies three direct determinants of behavioral intention to use technology (PE, EE, SI), two direct determinants of technology use (behavioral intention and facilitating conditions), and four moderators including gender, age, experience, and voluntariness that would alter the effect of the determinants on intention and/or behavior (25).

**Figure 1:**
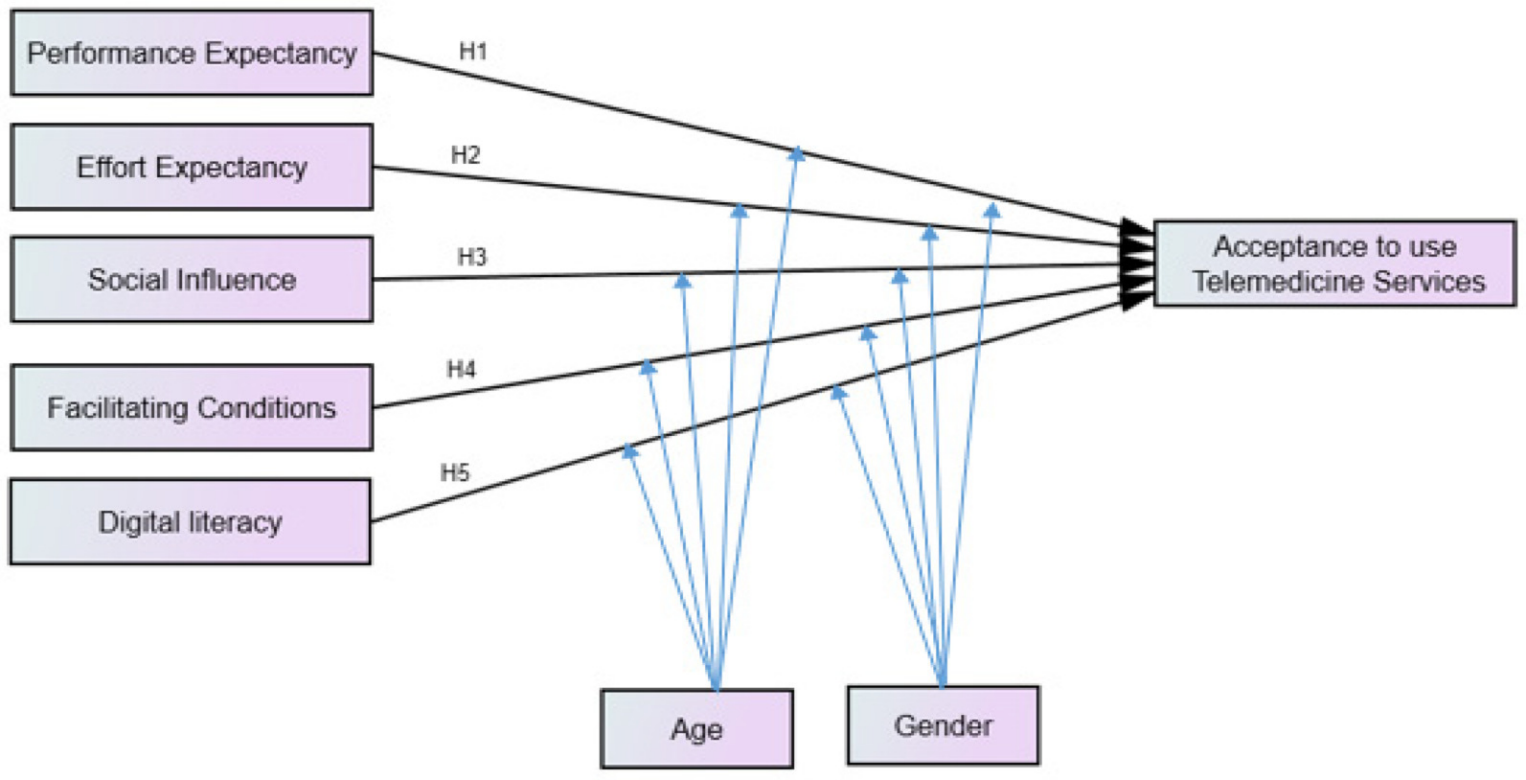
Conceptual framework for acceptance to use telemedicine services among healthcare professionals and its predictors, adapted UTAUT model. H*n*, is the hypothesis of the association between exogenous and endogenous variables. Adapted with permission from “The unified theory of acceptance and use of technologies (UTAUT)” by Mohammed Rouidi, Abdelmajid Elouadi, and Amine Hamdoune, licensed under CC BY-NC 4.0, and “Unified Theory of Acceptance and Use of Technology (UTAUT)” by Elske Ammenwerth, licensed under CC BY-NC 4.0.

This study considered the four original constructs and one additional construct, a total of five independent constructs (PE, EE, SI, FC, and DL), two moderators (age and gender), and behavior intention to use (“acceptance”) as a dependent construct (26, 27). Because the telemedicine services from Salale Digital Telemedicine Center have not been widely used and are in an infancy stage, and due to the unclear status of actual use of telemedicine services currently, the construct “actual use” which was considered as a dependent variable in the original UTAUT was not used in this study (25, 27). Actual usage behavior should be measured over the 6-month post-training (24). The effect of moderator experience was excluded from our model because most of the study participants might not have had prior telemedicine experience at the time the data were collected. In addition, the system was voluntary, users were not forced to use the system, and the moderator “voluntariness to use” was excluded from our model (28). Finally, the proposed theoretical model is presented in Figure 1.

**Figure 2:**
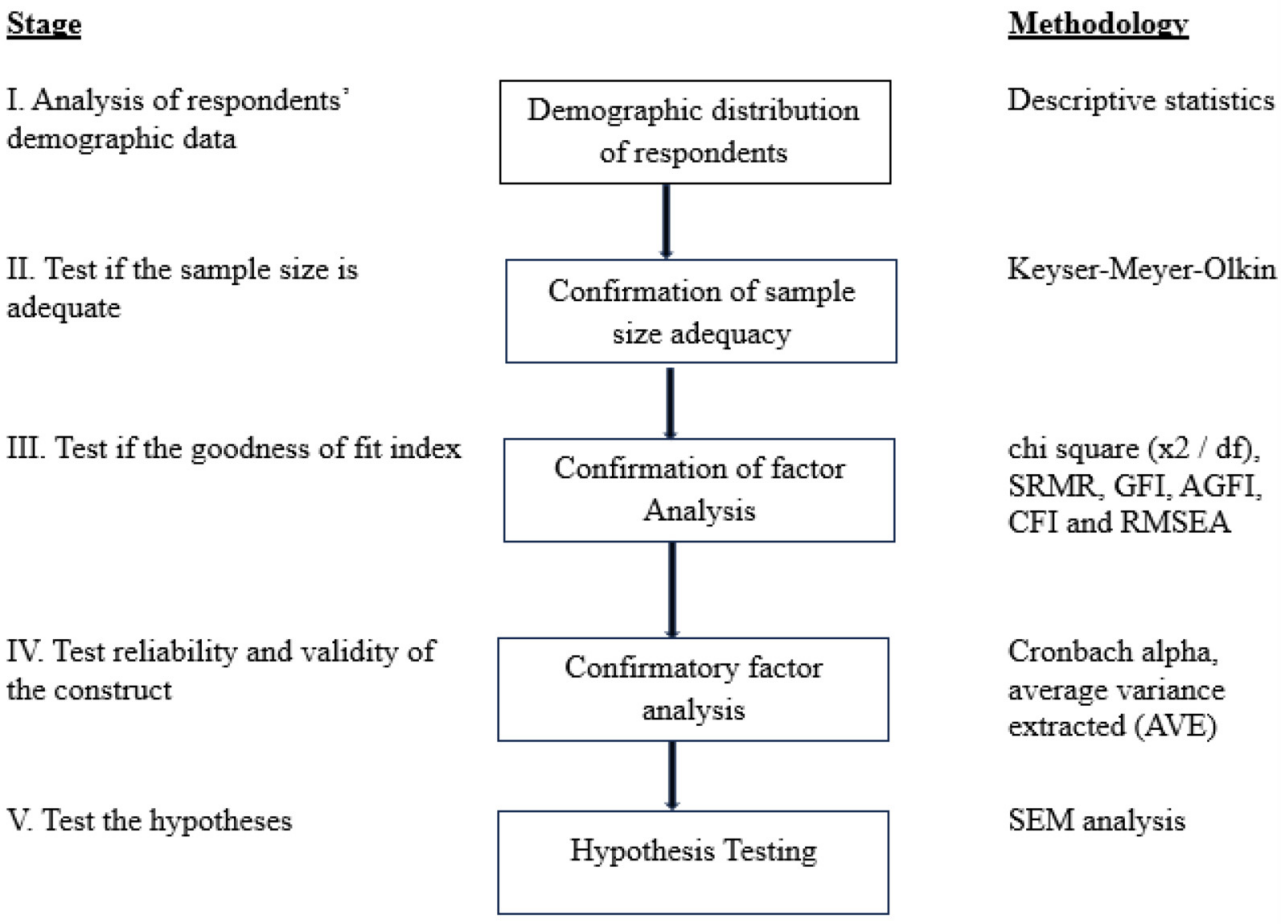
Data analysis and results framework.

## Factors affecting the acceptance to use telemedicine services

The theory was established on four original theoretical constructs performance expectancy (PE), effort expectancy (EE), social influence (SI), and facilitating conditions (FC), and one additional construct digital literacy (DL).

**Performance expectancy:** the degree to which an individual believes that using the system will help him or her to attain gains in job performance (24, 31). In this context, performance expectancy is the degree to which healthcare professionals believe that using telemedicine services will help him or her manage health and enhance job performance. According to prior studies, performance expectance is the main significant predictor of acceptance and has significant positive effects on behavioral intention (24, 32). A study conducted in South Korea using the UTAUT model found that performance expectancy has positive effects on behavioral intention to use telemedicine service (*p* < 0.05) (33). However, studies conducted in Iran (28), Tanzania (34), and Ethiopia during COVID-19 (35) showed that there was no significant relationship between performance expectancy and behavioral intention. Therefore, to test the effect of PE on acceptance to use (ATU), the following hypothesis has been proposed:

✓ H1: Performance expectancy has positively influenced healthcare professionals' acceptance to use telemedicine services.

**Effort expectancy:** the degree of ease associated with the use of the system (31). In this context, effort expectancy is the degree of easy-to-use telemedicine services by accessing the Salale Digital Telemedicine Center. Several studies have shown that the relationship between the construct effort expectancy and behavioral intention was significant (36, 37). Moreover, the study conducted in Ethiopia found that effort expectancy was a significant predictor of healthcare professionals' acceptance of telemedicine (35). To test the effect of EE on acceptance to use (ATU), the following hypothesis has been proposed:

✓ H2: Effort expectancy has positively influenced healthcare professionals' acceptance to use telemedicine services.

**Social influence:** the degree to which an individual perceives that important others believe he or she should use the new system (31). According to studies conducted in the United States (27), Southeast Michigan (38), Saudi Arabia (36), Korea (37), and Ethiopia on the use of telemedicine (39), there was a significant relationship between social influence and behavioral intention. In contrast, studies conducted in the Netherlands (40), Nigeria (5), Tanzania (34), and Ethiopia (35) showed that the relationship between social influence and behavioral intention was not significant. To test the effect of SI on acceptance to use (ATU), the following hypothesis has been proposed:

✓ H3: Social influence has positively influenced healthcare professionals' acceptance to use telemedicine services.

**Facilitating conditions:** the degree to which an individual believes that an organizational and technical infrastructure exists to support the use of the system (31).

Studies conducted in the United States (27), Spain (41), Korea (37), and Nigeria (5) showed that the relationship between facilitating conditions and behavioral intention was significant. Moreover, a study conducted in South Korea showed that FC is an influential factor in the acceptance of telemedicine services (33). On the other hand, a study conducted in Ethiopia (35) revealed that there was no significant association between facilitating conditions and behavioral intention. To test the effect of FC on acceptance to use (ATU), the following hypothesis has been proposed:

✓ H4: Facilitating conditions have positively influenced healthcare professionals' acceptance to use telemedicine services.

**Digital literacy (DL):** referring to a person's capacity to seek, evaluate, and communicate information using writing and other media across a range of digital platforms (36). In this model, to test the effect of DL on acceptance to use (ATU), the following hypothesis has been proposed:

✓ H5: Digital literacy has positively influenced healthcare professionals' acceptance to use telemedicine services.

## Moderating effects of acceptance to use telemedicine services

Moderator is a variable that can strengthen, diminish, negate, or otherwise alter the association between exogenous and endogenous variables, and it can also change the direction of this relationship. The moderators that were included in the UTAUT model are gender, age, experience, and voluntariness of use (31). The gender and age moderators were described here.

## Moderating effect of gender

Several studies have shown that gender had a moderating influence on the relationship between social influence and behavioral intention (33, 42) and is significant for the female group, indicating that women are more likely to be influenced by the opinions of others when deciding to use a new technology (36). A study conducted in South Korea using the UTAUT model reveals that the path between PE and behavioral intention to use telemedicine service is stronger for females than for males (33). In this model, to test the effect of the gender moderator on acceptance to use (ATU), the following hypothesis has been proposed:

✓ H6: The influence of performance expectancy (PE) on healthcare professionals' acceptance to use telemedicine services has been moderated by gender.
✓ H7: The influence of effort expectancy (EE) on healthcare professionals' acceptance to use telemedicine services has been moderated by gender.
✓ H8: The influence of social influence (SI) on healthcare professionals' acceptance to use telemedicine services has been moderated by gender.
✓ H9: The influence of facilitating conditions (FC) on healthcare professionals' acceptance to use telemedicine services has been moderated by gender.
✓ H10: The influence of digital literacy (DL) on healthcare professionals' acceptance to use telemedicine services has been moderated by gender.

## Moderating effect of age

According to a study conducted in South Korea using the UTAUT model, the path between performance expectancy and behavioral intention to use telemedicine services is stronger for the younger group than the older group (33). A study conducted in Canada showed that older age healthcare professionals are less comfortable using technology and are more resistant to it (42). However, several studies showed that age did not moderate the relationships between the independent variables and behavioral intention (36, 37, 43). In this model, to test the effect of the age moderator on acceptance to use (ATU), the following hypothesis has been proposed:

✓ H11: The influence of performance expectancy (PE) on healthcare professionals' acceptance to use telemedicine services has been moderated by age.
✓ H12: The influence of effort expectancy (EE) on healthcare professionals' acceptance to use telemedicine services has been moderated by age.
✓ H13: The influence of social influence (SI) on healthcare professionals' acceptance to use telemedicine services has been moderated by age.
✓ H14: The influence of facilitating conditions (FC) on healthcare professionals' acceptance to use telemedicine services has been moderated by age.
✓ H15: The influence of digital literacy (DL) on healthcare professionals' acceptance to use telemedicine services has been moderated by age.

## Methods

### Study design and setting

An institutional-based cross-sectional study design was conducted from 3 April to 1 May 2023 at public hospitals of the North Shewa Zone. North Shewa is one of the zones of the Oromia Regional State in Ethiopia. Fiche is the capital city of the zone, which is located 114 km distant from Addis Ababa, the capital city of Ethiopia. According to the 2007 Central Statistics Agency (CSA) report, the zone had a total population of 1,445,993, approximately 724,894 males and 721,099 females (44). In the North Shewa Zone, there are five public hospitals providing healthcare services for the population, namely, Salale University Comprehensive Specialized Hospital, Kuyu General Hospital, Dera Primary Hospital, Sheno Primary Hospital, and Muka Turi Primary Hospital.

### Study participants and sample size determination

All healthcare professionals working in all public hospitals of the North Shewa Zone were considered as the source population. All healthcare professionals available at the public hospitals during the data collection period were considered as the study population. Healthcare professionals who have <6 months of work experience at the time of the data collection period were excluded.

In this study, the researchers resolved the parameter identification process and evaluated whether the prior factor loadings of each item on their respective factors were estimated or identified using under-identified, just-identified, and overidentified. The number of distinct elements in the structural equation system (∑) or available inputs needs to be greater than the number of free parameters to be estimated to proceed with the given model. Therefore $\varepsilon =\frac{k(k+1)}{2}$ where *k* is the number of observed indicator variables in the model, which is 21 in this model. As a result, the number of distinct elements was $\varepsilon =\frac{21(21+1)}{2}=231$ distinct elements or the number of information, with each of these distinct elements having a corresponding equation. By considering the rules to estimate free parameters, 57 free parameters were considered in this hypothetical model.

To know whether the model is under, just, or overidentified, the degree of freedom (DF) is calculated as the number of distinct elements minus the number of free parameters to be estimated.

Degreeoffreedom=samplemoment−freeparameters=231−57=174

This implies that the above model was structurally overidentified model since DF > 0. It is recommended to use 5–20 samples per variable of free to be estimated (45). Therefore, considering 57 free parameters as a rule of thumb of 10 samples per indicator variable, a non-response rate of 10% was considered for the study; a 627 sample size was finally considered.

### Sampling procedure

Five hospitals (Salale University Comprehensive Specialized Hospital, Kuyu General Hospital, Dera Primary Hospital, Sheno Primary Hospital, and Muka Turi Primary Hospital) were included in our study. Each hospital was proportionally allocated based on the number of healthcare professionals it has. The study participants were chosen through simple random sampling by computer-generated method from each hospital.

## Study variables

### Dependent variable

Acceptance to use telemedicine services was the dependent variable which was dichotomized into two categories, i.e., accepted and not accepted.

### Independent variables

The independent variables were healthcare providers’ sociodemographic factors (age, gender, work experience, marital status, educational level, professional category, and type of health facility) and UTAUT model constructs, such as performance expectancy, effort expectancy, social influence, facilitating conditions, and digital literacy.

### Operational definition

**Acceptance to use telemedicine services:** an individual's psychological state with regard to his or her voluntary or intended use of a telemedicine technology (29, 46). In this case, acceptance to use was operationalized based on healthcare professionals’ responses to three questions measured on a five-point Likert scale. Participants who scored at or above the were categorized as having accepted the use of telemedicine, whereas those scoring below the median were categorized as not having accepted it (47).

**Performance expectancy:** the degree to which an individual believes that using the system will help him or her to attain gains in job performance (24, 31), This was measured with a five-point Likert scale of four questions.

**Effort expectancy:** the degree of ease associated with the use of the system (31). This was measured with a five-point Likert scale of four questions.

**Social influence:** the degree to which an individual perceives that important others believe he or she should use the new system (31). This was measured with a five-point Likert scale of three questions.

**Facilitating conditions:** the degree to which an individual believes that an organizational and technical infrastructure exists to support the use of the system (31). This was measured with a five-point Likert scale of four questions.

**Digital literacy:** referring to a person's capacity to seek, evaluate, and communicate information using writing and other media across a range of digital platforms (36). This was measured with a five-point Likert scale of three questions.

### Data collection tools and procedure

In this study, we used a questionnaire that was adapted from the original instrument developed by Venkatesh et al.'s study and several relative studies regarding the UTAUT model (24, 38, 48, 49). The questionnaire consists of two sections. Section 1 focuses on healthcare professionals’ demographic information, and Section 2 contains 21 statements that symbolize the constructs included in the UTAUT model, and the questions were measured using a five-point Likert scale with anchors of “strongly agree” to “strongly disagree,” with 1 denoting strongly disagree and 5 denoting strongly agree (23, 50, 51). A structured questionnaire was initially developed in the English language. As the participants were healthcare professionals and their day-to-day work activity was in English language, data were collected using a self-administered structured questionnaire in English version by five data collectors who have good English communication. Two MPH holder health professionals who have experience in research work supervised the data collection process. Data collectors and supervisors received a 2-day training on the research's goal and how to gather data. Before the survey, trained data collectors explained to the respondents about telemedicine services started at Salale Comprehensive Specialized Hospital to help them understand the significance of the survey questions, and they either agreed or refused to take part in the study. Any respondent who did not give their oral consent was thanked for their time. Finally, the data collectors conducted the data collection with those who had given their consent.

### Data quality control

To control the quality of data, a 2-day training was given for data collectors and supervisors about the study's purpose, data collection techniques, data collection tools, respondent approach, data confidentiality, and respondent rights before the actual data collection. Although a questionnaire was a standard tool, the instrument was pretested for its reliability and construct validity with 31 (5%) of the study participants among healthcare professionals at Chancho Hospital before actual data collection. As a result, the reliability of latent variables was above the threshold values (0.7) and Cronbach's alpha (0.754–0.891). However, a few changes and appropriate wording choices were made afterward, and actual data collection was begun based on the findings of the pretest. Continuous supervision was made to control the data collection procedure by supervisors to examine the completeness and accuracy of the surveys every day. After data collection, questionnaires were reviewed and checked for completeness, and the data were cleaned and cross-checked for errors and missed values and corrected if any errors were identified.

### Data processing and statistical analysis

To process the data, the filled-out and completed questionnaires were checked manually for completeness, and the data were coded and entered into EpiData version 4.6 and exported to SPSS version 25 software to estimate the descriptive statistics of demographic variables and proportion of acceptance to use telemedicine services. Structural equation modeling (SEM) with AMOS software version 21 was employed to assess the relationship between different latent and observable variables.

AMOS was used to perform confirmatory factor analysis (CFA) with standardized values to test the measurement mode. The normality of observations was the first and most important assumption before building the model and checking its fit indices. The assumption of multivariate outlier detection using Mahalanobis d-squared was checked, data normality was assessed using multivariate kurtosis <5, and the critical ratio (CR) was between −1.96 and +1.96. The maximum likelihood (ML) technique of approximation was used to get the estimates of parameters when the study's variable exhibits normal. But if this assumption is violated, it is recommended to use estimation methods such as bootstrapping methods (52).

The sample size to be considered in SEM is large. The minimum sample size that should be utilized in the SEM method is at least 10 times the number of parameters that can be estimated in the model (53). In the SEM, it is assumed that there is no relationship between the independent variables, as the correlation between exogenous constructs should be <0.8. Also, multicollinearity was checked using tolerance >0.1 and variance inflation factors (VIF) < 10 (54).

A correlation between constructs and factor loadings for each item was tested as part of CFA to check that the value of the factor loading for each item should be >0.5 (55). If the model fit indices were below the cutoff point (0.5) or there was model misspecification, we were either to delete the item that was below the cutoff point (0.5) or use a high value of modification indices to enhance model fit indices until the model was fitted with a threshold value of maximum 4 times (55). The chi-square ratio (<3), goodness-of-fit index (GFI > 0.9), adjusted goodness-of-fit index (AGFI > 0.8), root-mean-square error of approximation (RMSEA < 0.08), and root-mean-square of standardized residual (RMSR < 0.08) were used to assess the model’s goodness of fit. Construct reliability and validity were evaluated to determine the extent to which a variable or combination of variables was consistent in what it wanted to measure and to evaluate how effectively the selected construct item measured the construct (Figure 2). Cronbach's alpha test and composite reliability were used to assess internal reliability with the recommended threshold value of 0.7 and above (56). The scale items' convergent validity was determined using the average variance extracted (AVE) method, with values above the 0.50 threshold. The Fornell and Larcker criterion was used to determine discriminant validity, and it was supported if the square root of AVE for a construct was greater than its correlation with the other constructs in the study (55).

The square multiple correlation (*R*^2^) was used to report the magnitude of variances in endogenous latent variables explained by exogenous variables. To test the structural model, the standardized path coefficient was used to measure the relationship between exogenous and endogenous variables, as well as 95% confidence intervals, and *p* < 0.05 was used to declare a statistically significant association. The moderator can be a continuous or categorical variable, which can alter the relationship between the independent and dependent variables through interaction effects and multiple group analysis (47). In this study, because gender (male, female) and age (<30- and ≥30-year age group) were dichotomized as binary, the moderating effects of predictors among the hypothesized paths within the core research model were tested using multiple group analysis. The chi-square difference and *p*-value between the unconstrained and constrained models were estimated to determine the effect of the moderator.

## Results

### Sociodemographic characteristics of healthcare professionals

A total of 627 questionnaires were distributed to participants, and 601 questionnaires were retrieved from participants. Thus, 601 (95.85% response rate) respondents were finally used in this study. The median age of the respondents ranged from 23 to 54 years, with a median age of 30.0 ± 5 interquartile range. Majority of the respondents were male 398 (66.2%). According to their profession, 276 (45.9%) were nurses. The median work experience of the respondents ranged from 1 to 25 years, with a median age of 5.0 ± 4 interquartile range. The educational level of participants was observed mainly for the bachelor's holder category 379 (63.1%) followed by 167 (27.8%) for master and above (Table 1).

**Table 1:** Sociodemographic characteristics of healthcare professionals at public hospitals in North Shewa Zone of Oromia Regional State, Ethiopia (2023) (*n* = 601).

Characteristics	Category	Frequency (*n*)	Percentage (%)
Gender	Male	398	66.2%
	Female	203	33.8%
Marital status	Single	222	36.9%
	Married	372	61.9%
	Others	7	1.2%
Profession	Physicians	124	20.6%
	Nurses	276	45.9%
	Midwife	91	15.1%
	Pharmacy	36	6.0%
	Others*	74	12.3%
Educational level	Diploma	55	9.2%
	Degree	379	63.1%
	Master and above	167	27.8%
Facility type	Primary hospitals	257	42.8%
	General hospital	119	19.8%
	Comprehensive specialized hospital	225	37.4%

Others^*^: laboratory, radiology, public health, anesthesia, optometry, and psychiatry.

### Acceptance to use telemedicine services

**Figure 3:**
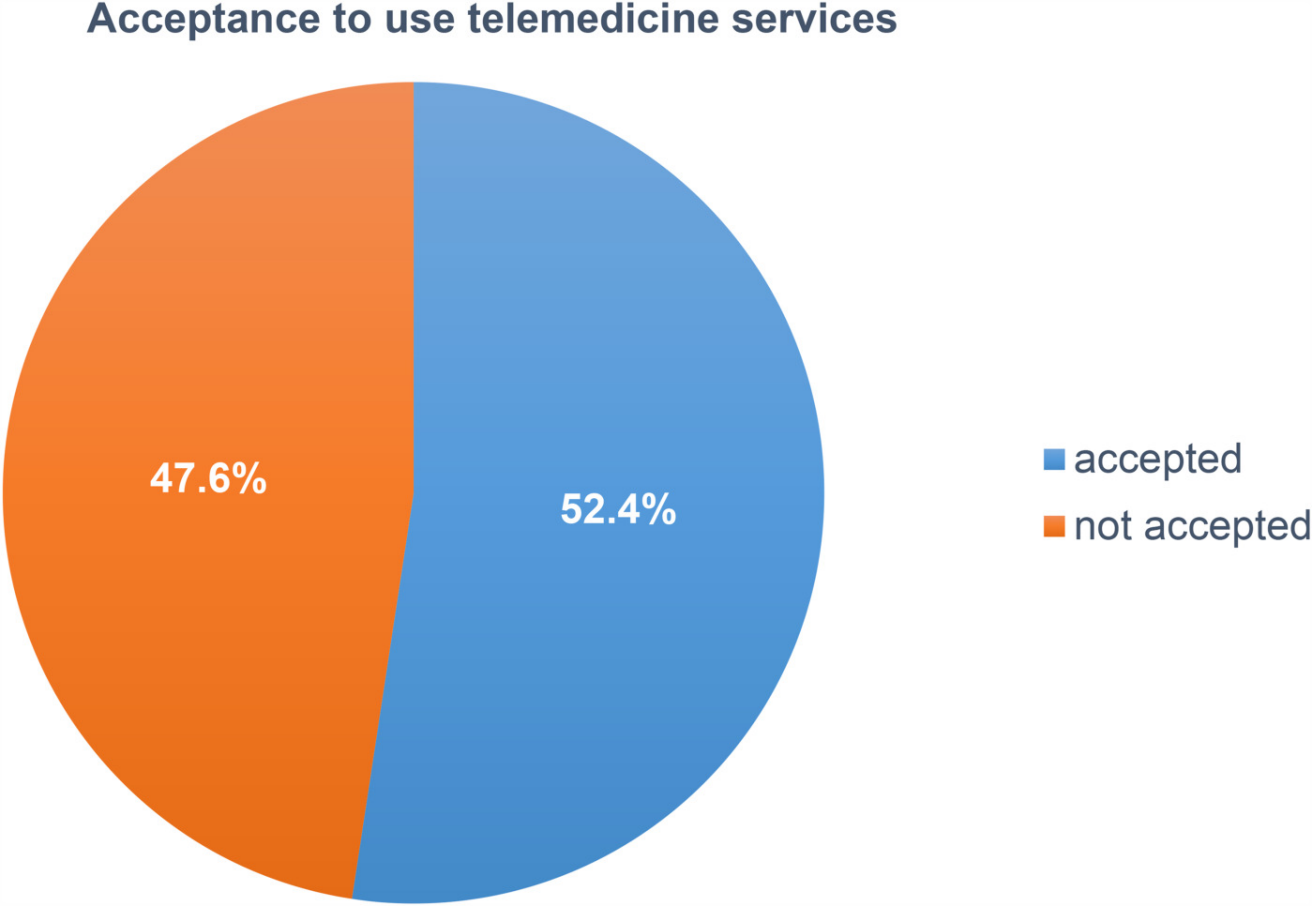
Proportion of healthcare professional's acceptance to use telemedicine services at public hospitals in North Shewa Zone of Oromia Regional State, Ethiopia (2023).

Accordingly, 315 (52.4%) (95% CI: 48.3–56.5) of healthcare professionals accepted to use the offered telemedicine services with a median score of 12 [interquartile range (10–13)] and minimum and maximum scores of 3 and 15, respectively (Figure 3).

### Measurement model assessment

**Figure 4:**
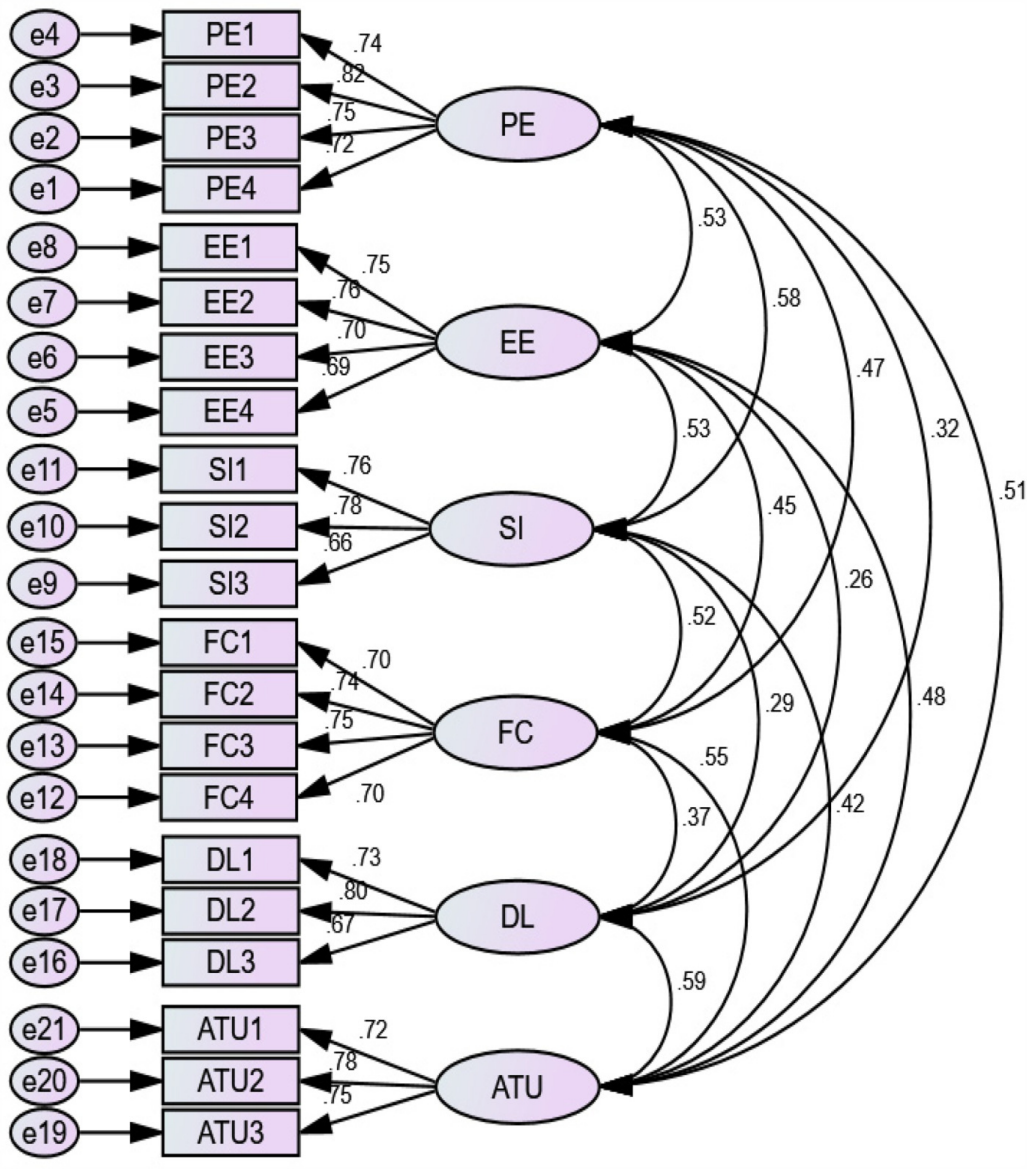
Confirmatory factor analysis of acceptance to use telemedicine services at public hospitals in North Shewa Zone of Oromia Regional State, Ethiopia (2023).

Confirmatory factor analysis (CFA) was used to evaluate the measurement model by examining the indicators' model fit, internal consistency, convergent validity, and discriminant validity. The multivariate kurtosis value in this study was >5 (kurtosis = 35.44), and the multivariate critical ratio (CR) did not fall within the range of −1.96 to +1.96 (CR = 13.97), indicating that our data were not normally distributed. Assuming a normal distribution was utilized in this instance, the non-parametric test of bootstrapping methods (57) aids non-normal data by resampling the data and estimating the significance of the path coefficients, standard errors, and confidence intervals. Thus, 5,000 bootstrap samples were used in AMOS, with a 95% bias-corrected confidence interval (58). The CFA is shown in Figure 4.

### Goodness-of-fit index

This study used seven measures to assess the goodness of fit of the CFA: chi-square (*χ*^2^/DF), standardized root-mean-squared residual (SRMR), goodness-of-fit index (GFI), adjusted goodness-of-fit index (AGFI), normative fit index (NFI), comparative fit index (CFI), and root-mean-square error of approximation (RMSEA). Thus, the overall values were within acceptable thresholds and implied that the model was fit for estimation (Table 2).

**Table 2:** Overall goodness-of-model fit summary of the research.

Matrix indices	Cutoff point	Authors	Result	Interpretation
(*χ*^2^/DF^)^	<3	Bentler (1990)	1.829	Excellent
RMSR	<0.08	Byme (2001)	0.029	Excellent
GFI	>0.9	Chau (1997)	0.950	Excellent
AGFI	>0.8	Chau (1997)	0.934	Excellent
NFI	>0.9	Bentler and Bonett (1990)	0.939	Excellent
CFI	>0.9	Bentler (1990)	0.971	Excellent
RMSEA	<0.08	Byme (2001)	0.037	Excellent

(*χ*^2^/DF): chi-square divided by degrees of freedom.

### Reliability and validity of the construct

A CFA was used to evaluate the measurement models for the validity and reliability of constructs (59). To measure the internal reliability of the data, Cronbach’s alpha was evaluated against the standard threshold of 0.7. The Cronbach's alpha values for individual constructs ranged from 0.773 to 0.841, thus exceeding the threshold of 0.70. The range of composite reliability was varied from 0.776 to 0.844. As the calculated indices were all above the recommended threshold of 0.7 (60), strong internal reliability of data was supported.

Convergent validity was calculated using average variance extracted (AVE) and factor loadings. As a result, the AVE for all reflective constructs was above the proposed level of 0.5, ranging from 0.527 to 0.575, whereas the values for an individual factor loading of constructs were all above 0.50, ranging from 0.695 to 0.820, exceeding the recommended level. Hence, these findings showed that the proposed model's convergent validity and reliability were achieved (Table 3).

**Table 3:** Convergent validity between constructs for acceptance to use telemedicine services at public hospitals in North Shewa Zone of Oromia Regional State, Ethiopia (2023).

Constructs	Items	Factor loadings	Composite reliability (CR)	Average variance extracted (AVE)	Cronbach's alpha
Performance expectancy	PE1	0.736	0.844	0.575	0.841
	PE2	0.820			
	PE3	0.754			
	PE4	0.718			
Effort expectancy	EE1	0.749	0.817	0.528	0.817
	EE2	0.757			
	EE3	0.703			
	EE4	0.695			
Social influence	SI1	0.763	0.779	0.542	0.774
	SI2	0.782			
	SI3	0.657			
Facilitating conditions	Fc1	0.703	0.817	0.527	0.816
	FC2	0.743			
	FC3	0.755			
	FC4	0.703			
Digital literacy	DL1	0.729	0.776	0.538	0.773
	DL2	0.797			
	DL3	0.668			
Acceptance to use	ATU1	0.715	0.793	0.562	0.792
	ATU2	0.782			
	ATU3	0.750			

PE, performance expectancy; EE, effort expectancy; SI, social influence; FC, facilitating conditions; DL, digital literacy; ATU, acceptance to use; AVE, average variance extracted.

To confirm that reflective constructions differ from one another, the discriminant validity was determined using the square root of AVE in place of the matrix diagonal correlation coefficient. The measurements used in this investigation provided a solid confirmation of the structural model's discriminant validity, convergent validity, and unidimensionality. The Fornell–Larcker criterion was regarded for determining discriminant validity (61). According to this criterion, the square root of each construct's AVE should be greater than the squared correlation with any other construct. All of the constructs had AVE values that were higher than 0.50, ranging from 0.527 to 0.575. The square root of the AVE for each of the constructs (diagonal values), ranging from 0.726 to 0.758, was also higher than its highest correlation with any other constructs. As a consequence, the discriminant validity of the model's constructs was attained (Table 4).

**Table 4:** Discriminant validity between constructs using Fornell–Larcker criterion for acceptance to use telemedicine services at public hospitals in North Shewa Zone of Oromia Regional State, Ethiopia (2023).

Constructs	AVE	PE	EE	SI	FC	DL	BI
PE	0.575	0.758					
EE	0.528	0.526	0.727				
SI	0.542	0.584	0.530	0.736			
FC	0.527	0.469	0.453	0.520	0.726		
DL	0.538	0.325	0.262	0.288	0.370	0.733	
Acc	0.562	0.512	0.484	0.422	0.553	0.595	0.750

### Structural equation model assessment

Before SEM analysis was used to assess the hypotheses, collinearity had to be confirmed, along with the measurement model's validity and the absence of any significant correlations between exogenous constructs. The variance inflation factor (VIF) and tolerance were used to test multicollinearity. The values for tolerance and variance inflation factors should be >0.1 and <10, respectively. Thus, there was no multicollinearity problem among exogenous constructs (Table 5).

**Table 5:** Multicollinearity test between constructs for acceptance to use telemedicine services at public hospitals in North Shewa Zone of Oromia Regional State, Ethiopia (2023).

Constructs	Tolerance	VIF
PE	0.667	1.499
EE	0.712	1.404
SI	0.669	1.494
FC	0.727	1.376
DL	0.882	1.133

**Figure 5:**
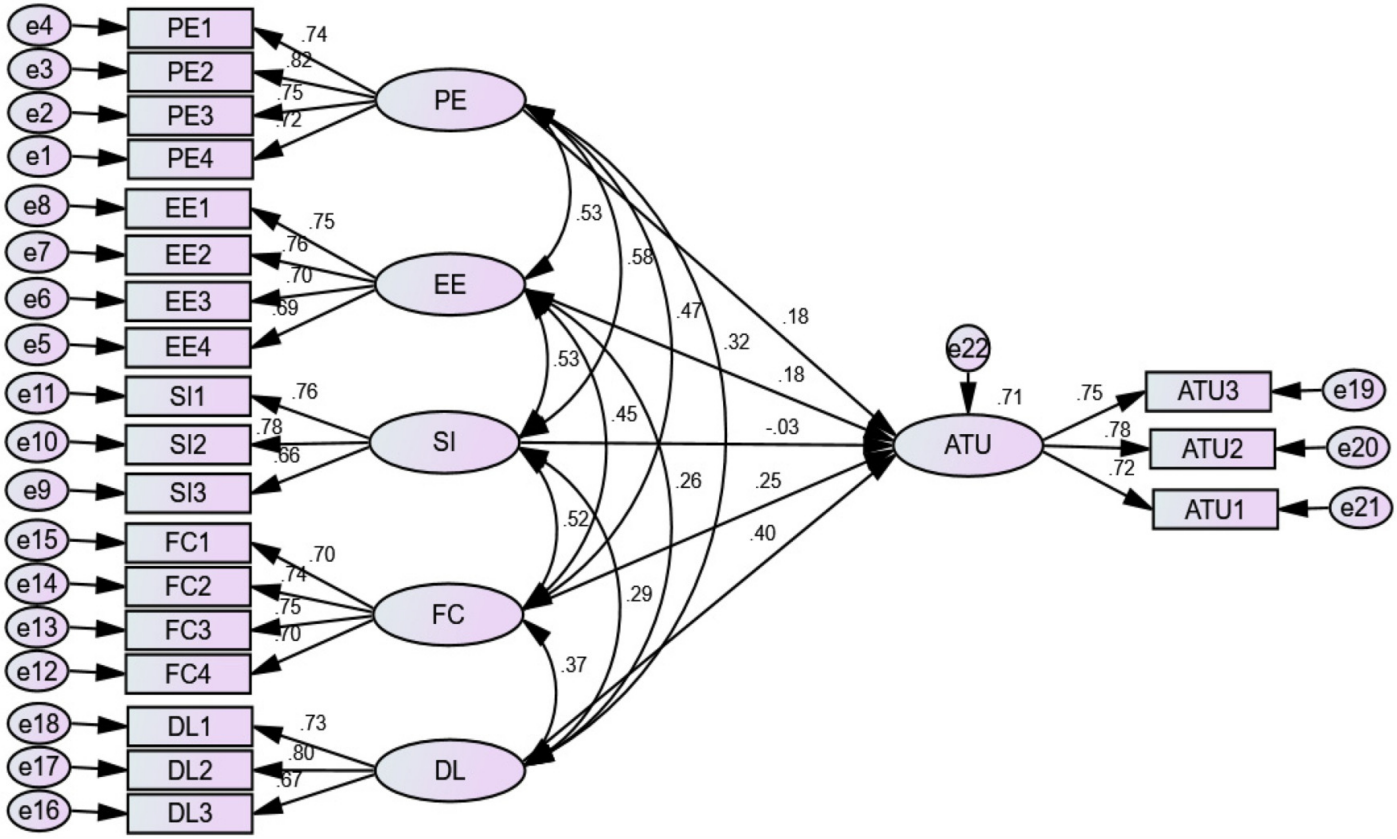
SEM analysis for predictors of acceptance to use telemedicine services at public hospitals in North Shewa Zone of Oromia Regional State, Ethiopia (2023).

The coefficient of determination (*R*^2^) was 0.71. This finding indicated that the predictors of acceptance to use telemedicine services explained 71% of the variance in acceptance to use telemedicine services (an endogenous construct). Thus, the proposed model had strong predictive power (Figure 5).

To ascertain the relationships between the constructs in the research model, a structural model was built. The path diagram in the results of the structural model analysis (Table 6) showed that performance expectancy, effort expectancy, facilitating conditions, and digital literacy had a significant positive effect on an acceptance to use telemedicine services, while the construct social influence did not. Accordingly, the H1, H2, H4, and H5 were accepted, and on the other hand, the H3 was rejected.

**Table 6:** SEM analysis of predictors of acceptance to use telemedicine services among healthcare professionals at public hospitals in North Shewa Zone of Oromia Regional State, Ethiopia (2023).

Path	Estimate	SE	CR	*p*-value	95% confidence interval		Result
					Lower	Upper	
PE ATU	0.184	0.057	3.186	0.001**	0.067	0.308	Supported
EE ATU	0.183	0.059	3.319	***	0.062	0.300	Supported
SI ATU	−0.028	0.064	−0.463	0.644	−0.155	0.092	Not supported
FC ATU	0.249	0.061	4.460	***	0.138	0.351	Supported
DL ATU	0.403	0.060	7.728	***	0.320	0.485	Supported

*** *p* < 0.001, **p < 0.01.

SE, standard error; CR, critical ratio.

The study result showed performance expectancy had a significant and direct positive effect on the healthcare professional's acceptance to use telemedicine services [*β* = 0.184, 95% CI: (0.067–0.308), *p* = 0.001]. This result indicates that H1 was supported and that an increase in healthcare professional's performance expectancy leads to an increase in acceptance to use telemedicine services. Effort expectancy also had a significant direct effect on the acceptance to use telemedicine services [*β* = 0.183, 95% CI: (0. 0602–0. 300), *p* < 0.001]. This indicates that the more healthcare professionals view telemedicine as an easy-to-use technology, the more they will be motivated to utilize these telemedicine services. Thus, H2 was supported.

Similarly, the facilitating condition had a significant direct effect on acceptance to use telemedicine services [*β* = 0.249, 95% CI: (0. 138–0. 351), *p* < 0.001]. This result showed that an improved organizational facilitating condition or infrastructure leads to an increase in healthcare professionals’ acceptance to use telemedicine services. Consequently, H4 was supported.

Digital literacy had the most significant direct effect on acceptance to use telemedicine services [*β* = 0.403, 95% CI: (0. 320–0. 485), *p* < 0.001]. Healthcare professionals are more willing to accept telemedicine services as their level of digital literacy increases. In this case, the H5 was supported. However, the construct social influence [*β* = −0.028, 95% CI: (−0.155 to 0.092), *p* = 0.644] had no direct effects on an acceptance to use telemedicine services. This indicates that healthcare professionals might not accept new technology through the influence of others. Thus, H3 was not supported.

### Moderation effects

The association between performance expectancy, effort expectancy, social influence, facilitating conditions, and digital literacy with healthcare professionals' acceptance to use telemedicine services was examined, along with the moderating influences of gender and age. To test the moderators, two model comparisons were computed using unconstrained and constrained models. While the constrained model assumption suggests that the variable has a similar effect on influencing the relationship between the exogenous and endogenous variables, the unconstrained model assumption showed that there is a moderator or static difference in the given variable to influence the exogenous and endogenous variables. The proposed moderator variable was confirmed as a moderator if the significant difference between the two models was determined to be significant (*p* < 0.05 or chi-square difference >5) (62).

To the test hypotheses [H6–H15], we first performed an individual estimation for gender (males and females) and age [younger group (<30 years) and older group (≥30 years)] (63) and followed by a multigroup analysis to determine the gender and age moderator to examine whether the strengths of the paths were different between sub-groups. Unfortunately, we found that there were no gender-specific significant differences in the acceptance to use telemedicine services along the paths of performance expectancy, effort expectancy, social influence, facilitating conditions, and digital literacy. Thus, H6–H10 were not supported, as shown below in Table 7.

**Table 7:** Moderating effects of gender for acceptance to use telemedicine services among healthcare professionals at public hospitals in North Shewa Zone of Oromia Regional State, Ethiopia (2023).

Hypothesis	Moderator	Path coefficient	*p*-value	Model test (constrained and unconstrained)		Result
	Gender			*Δχ* ^2^	*p*-value	
PE	Male	0.217	***	1.919	0.166	Not supported
	Female	0.119	0.041			
EE	Male	0.225	***	0.011	0.915	Not supported
	Female	0.218	***			
SI	Male	−0.090	0.046	0.199	0.655	Not supported
	Female	−0.055	0.397			
FC	Male	0.272	***	1.693	0.193	Not supported
	Female	0.368	***			
DL	Male	0.531	***	0.000	0.992	Not supported
	Female	0.530	***			

*** *p* < 0.001.

Additionally, we found that the path between performance expectancy, effort expectancy, and social influence on acceptance to use telemedicine services was not significantly different between individuals by age, while facilitating conditions and digital literacy were significant. Therefore, H11–H13 were not supported, as shown in Table 8. The relationship between FC and acceptance to use telemedicine services was positively moderated by age and significantly stronger for younger group respondents (*β* = 0.400, *p* < 0.001) compared with the relationship for the older group (*β* = 0.237, *p* < 0.001). Thus, H14 was supported.

**Table 8:** Moderating effects of age for acceptance to use telemedicine services among healthcare professionals at public hospitals in North Shewa Zone of Oromia Regional State, Ethiopia (2023).

Hypothesis	Moderator	Path coefficient	*p*-value	Model test (constrained and unconstrained)		Result
	Age			*χ* ^2^	*p*-value	
PE	<30	0.165	***	0.465	0.495	Not supported
	≥30	0.209	***			
EE	<30	0.237	***	0.385	0.535	Not supported
	≥30	0.195	***			
SI	<30	−0.125	0.036	0.778	0.378	Not supported
	≥30	−0.057	0.227			
FC	<30	0.400	***	5.422	0.020	Supported
	≥30	0.237	***			
DL	<30	0.456	***	5.462	0.019	Supported
	≥30	0.598	***			

*** *p* < 0.001.

The relationship between digital literacy and acceptance to use telemedicine services was positively moderated by age and significantly stronger for the older group (≥30 years) (*β* = 0.598, *p* < 0.001) compared with the younger group (<30 years) (*β* = 0.456, *p* < 0.001). Consequently, H15 was supported.

## Discussion

This study aimed to investigate the acceptance to use telemedicine services and its predictors among healthcare professionals in public hospitals, which was examined using the UTAUT model. According to our finding, the proportion of healthcare professionals on acceptance to use telemedicine services was 315 (52.4%). This finding showed that approximately half of the healthcare professionals accepted the offered telemedicine services to provide healthcare for their patients and to improve their productivity. Although the finding is only approximately half, the offered telemedicine technology is a promising one for the future because the national digital blueprint has identified potential high-impact digital health interventions, particularly provider-to-provider telemedicine, that can be effectively and easily scaled up in the next 10 years (64). Additionally, Ethiopia currently has a low level of emerging technology development (65). Moreover, telemedicine technology is still in its infancy stage of development in Ethiopia and other sub-Saharan African countries (66), and considering this, our finding on the acceptance to use telemedicine services among healthcare professionals was promising for the future.

The predictors of acceptance to use telemedicine services explained 71% (*R*^2^ = 0.71) of the variance in an endogenous construct, which indicated that the UTAUT was a substantial predictive model of healthcare professionals' acceptance to use telemedicine services. In this study, performance expectancy (PE) had a direct effect on healthcare professionals’ acceptance to use telemedicine services (*β* = 0.184, *p* = 0.001). This indicates that healthcare professionals have focused on the usefulness of telemedicine services in their daily work and are willing to increase their productivity by making it easier to diagnose difficult cases and improve patient care. This finding was consistent with some studies conducted in South Korea (*β* = 0.345, *p* < 0.01) (33), Slovenia (*β* = 0.25, *p* < 0.001) (67), Malaysia (*β* = 0.207, *p* < 0.05) (68), and Nigeria (*β* = 0.0901, *p* < 0.05) (5). This finding implicated that healthcare professionals are likely to accept a telemedicine technology (Salale Digital Telemedicine Center) when it is considered to be useful to their practice which might enhance their job performance and productivity. The possible explanation could be also that telemedicine can provide value through the redistribution of medical expertise, the incorporation of interdisciplinary providers' contributions, and the creation of new educational possibilities (69). In contrast, this study was contradicted by the study conducted on telemedicine during COVID-19 in Ethiopia (*β* = −0.11, *p* > 0.05) (35). This discrepancy may be accounted for by the fact that the investigation was conducted using online surveys.

The result of this study also showed that effort expectancy (EE) has a significant association with acceptance to use telemedicine services (*β* = 0.183, *p* < 0.001). According to this finding, healthcare professionals who believe in the ease of use of the provided telemedicine services can easily understand and accept such technologies. This result was in accordance with the findings of previous studies conducted in Pakistan (*β* = 0.148, *p* < 0.05) (70), Slovenia (*β* = 0.52, *p* < 0.01) (67), Ghana (*β* = 3.155, *p* < 0.01) (71), and Ethiopia (*β* = 0.27, *p* < 0.05) (35). The possible explanation could be that the users are motivated to accept these services as they perceive telemedicine as an easy technology (70), and healthcare professionals can easily carry out their duties by employing telemedicine services.

Another finding of this study showed that the social influence (SI) was insignificant in predicting healthcare professionals' acceptance to use telemedicine services (*β* = −0.028, *p* = 0.644). This finding indicates that healthcare professionals are highly motivated to accept telemedicine irrespective of the opinions of important others. This finding was congruent with studies conducted in Slovenia (*β* = −0.03, *p* > 0.05) (67), Nigeria (*β* = −0.0902, *p* > 0.05) (5), and Ethiopia (*β* = −0.18, *p* > 0.05) (35). This finding was consistent with the viewpoint healthcare professionals' telemedicine technology acceptance decisions might vary depending on their levels of autonomy, be made independently from other members of the healthcare team, and be evaluated in light of their routine clinical tasks and services (49). However, this finding was inconsistent with a study conducted in Pakistan (70); this might be due to population differences, as their study was conducted in a community where social influence is more important. Additionally, this is inconsistent with the study conducted in India (*β* = 0.690, *p* < 0.001) (72); it might be because cultural differences can play a role in how people perceive the opinions of others who are important to them (36). This disparity between our proposed hypothesis and the finding may be due to the possibility that the characteristics of our sample, such as demographics, professional background, or familiarity with telemedicine, may have played a role in diminishing the observed effect of social influence. Additionally, healthcare professionals' acceptance to use telemedicine services is influenced by a variety of factors other than social influence, such as performance expectancy, effort expectancy, facilitating conditions, or digital literacy with telemedicine.

This finding has also illustrated that facilitating condition (FC) has a direct effect on healthcare professionals’ acceptance to use telemedicine services (*β* = 0.249, *p* < 0.001). This result indicated that organizational conditions matter in shaping the acceptance of telemedicine services among healthcare professionals. This finding was consistent with studies conducted in Slovenia (*β* = 0.13, *p* < 0.01) (67). This might be because organizations identify important factors that need to be considered carefully when telemedicine technology is implemented, and healthcare practitioners might also have better access to facilitating conditions that make it easier for them to use the telemedicine system. The availability of resources, support, and knowledge are necessary to promote the acceptability of telemedicine services, and it is a prerequisite that medical professionals be able to monitor and provide feedback using adequate healthcare infrastructure (70). This is because healthcare professionals were highly influenced to accept telemedicine services based on their organizational and infrastructural preconditions. However, this study was inconsistent with the study conducted in Ethiopia (*β* = −0.18, *p* > 0.05) (35). This discrepancy may be attributed to the sample size and/or the fact that their research data were gathered via online surveys, which makes it possible that the healthcare professionals did not have sufficient knowledge about telemedicine technology.

This finding also showed that a strong relationship exists between digital literacy (DL) and acceptance to use telemedicine services (*β* = 0.403, *p* < 0.001), which was larger than the effects of other predictors on acceptance. This suggests that healthcare professionals who know about different digital technologies and need to use them for their jobs are likely to accept telemedicine technology. This study was consistent with studies conducted in Saud Arabia (*β* = 0.31, *p* < 0.001) (36) and Ethiopia (*β* = 0.087, *p* = 0.029) (62). This indicates that healthcare professionals who acquire adequate skills to search, understand, evaluate, and use health information available online can accept technology for better healthcare outcomes (73). Therefore, this finding suggests that the digital literacy predictor is a crucial factor influencing healthcare providers' acceptance to use telemedicine services. Accordingly, developing digital literacy training programs that focus on enhancing the digital literacy skills of healthcare workers at all levels, incorporating digital literacy as a fundamental part of healthcare workers' continuing professional development curriculum, and establishing mentorship programs where more tech-savvy professionals help their colleagues to develop digital skills might be strategies on how to improve the digital literacy of healthcare professionals. The digital literacy predictor is also implicated as an additional predictor for the theoretical understanding of the UTAUT model. Based on this, the finding might be used as input to policymakers, as they should encourage healthcare institutions to provide continuous digital literacy training to ensure healthcare professionals are proficient in using telemedicine platforms.

In this study, we also found that there was no significant difference between males and females in their acceptance to use telemedicine services. This outcome shows that telemedicine will be accepted regardless of a person's gender for use in their daily operations. This finding is supported by a study conducted in Saudi Arabia (74). There wasn’t much evidence to support the role of gender moderators in this study, despite the fact that they are crucial in the healthcare context (74). This study also found that the relationship between PE, EE, and SI with acceptance to use was not significant. This finding was supported by the study conducted in Saudi Arabia (43) and Australia (75).

Finally, we found that the relationship between facilitating conditions and healthcare professionals' acceptance to use telemedicine services was positively moderated by age (*β* = 5.422, *p* = 0.020). This effect was stronger for the younger group (<30 years). The possible explanation might be that younger healthcare professionals (<30 years) have the knowledge necessary to use telemedicine technology and they might get more technological support than the older age group (≥30 years). Moreover, the relationship between digital literacy and healthcare professionals' acceptance to use telemedicine services was positively moderated by age (*β* = 5.462, *p* = 0.019). This effect was stronger for the older group (≥30 years). A possible explanation could be that 90.7% of the respondents in the older group (≥30 years) have a BSc or above as their educational status.

## Conclusion

This study examined factors affecting healthcare professionals' acceptance to use telemedicine services. The findings of the study showed that the acceptance of the provided telemedicine services was found to be promising from the perspective of healthcare professionals. Our research also found that performance expectancy, effort expectancy, facilitating conditions, and digital literacy constructs have significant positive effects on healthcare professionals’ acceptance to use telemedicine, while social influence did not. Digital literacy appeared to be the most significant factor affecting healthcare professionals' acceptance to use telemedicine services, as its path coefficient was the highest. Facilitating conditions and digital literacy with acceptance to use were moderated by age. Since healthcare professionals have shown promising acceptance to use telemedicine services, healthcare administrators should invest in ongoing training for healthcare professionals to ensure they are at ease using the technology. To increase accessibility and expand the use of telemedicine, policymakers should make sure that the technological infrastructure needed for it is robust, reliable, and easy to access. Based on the promising outcome in our study setting, policymakers could scale the telemedicine services technology to other regions, and efforts should be made to streamline the integration of telemedicine technology into the workflows of healthcare with particular emphasis on underserved areas such as Ethiopia. This could involve coordinating efforts between local hospitals, regional health authorities, and the Ministry of Health.

## Data Availability

The original contributions presented in the study are included in the article/Supplementary Material, further inquiries can be directed to the corresponding author/s.
